# Central Diabetes Insipidus Following Steroid Replacement for Hypoadrenalism in Pembrolizumab-Induced Hypophysitis

**DOI:** 10.7759/cureus.107752

**Published:** 2026-04-26

**Authors:** Abdalrahman Alasmar, Su Su San, Thu Ta, Sunithi Elizabeth Mani

**Affiliations:** 1 Internal Medicine, Ungoofaaru Regional Hospital, Ungoofaaru, MDV; 2 Oncology, The Brunei Cancer Centre, Bandar Seri Begawan, BRN; 3 Radiology, Jerudong Park Medical Centre, Jerudong, BRN

**Keywords:** central diabetes insipidus (cdi), endocrinopathy, hypoadrenalism in old people, hypoadrenalism in the elderly, immunotherapy, pembrolizumab

## Abstract

Immune checkpoint inhibitors are increasingly associated with complex immune-related endocrine adverse events that may evolve dynamically over time. We report a rare case of pembrolizumab-induced hypophysitis complicated by central diabetes insipidus, which was unmasked following glucocorticoid replacement. A 68-year-old female patient receiving immunotherapy for lung adenocarcinoma presented with hyponatremia and secondary adrenal insufficiency, followed by abrupt severe hypernatremia after steroid initiation. Subsequent evaluation confirmed central diabetes insipidus, and treatment with desmopressin resulted in clinical and biochemical recovery. This case highlights the diagnostic challenges posed by overlapping pituitary dysfunction and emphasizes the importance of dynamic endocrine reassessment when electrolyte disturbances evolve during immunotherapy.

## Introduction

Immune checkpoint inhibitors (ICIs), including pembrolizumab, have significantly improved outcomes in a range of advanced malignancies by enhancing antitumor immune responses. However, their use is associated with immune-related adverse events affecting multiple organ systems, with endocrine complications among the most frequently reported [1,2].

Hypophysitis is a well-recognized endocrine complication of ICIs and most commonly presents with anterior pituitary hormone deficiencies, particularly secondary adrenal insufficiency [2,3]. In contrast, involvement of the posterior pituitary resulting in central diabetes insipidus (CDI) is rare and often overlooked [4,5]. CDI is characterized by impaired secretion of antidiuretic hormone, leading to excessive free-water loss and hypernatremia.

When adrenal insufficiency and CDI occur together, diagnosis may be challenging. Cortisol deficiency reduces renal free-water clearance by increasing antidiuretic hormone activity and impairing glomerular filtration, which can initially limit urine output and lead to dilutional hyponatremia. As a result, the typical features of CDI (polyuria and hypernatremia) may be masked. Following glucocorticoid replacement, restoration of renal free-water clearance can abruptly unmask underlying CDI, resulting in sudden polyuria and severe hypernatremia [6,7].

Although isolated cases of ICI-associated CDI have been reported, presentations in which CDI becomes clinically apparent only after steroid replacement remain uncommon [5,8]. This case illustrates the dynamic evolution of immune-related endocrine dysfunction in patients receiving ICIs and highlights the importance of careful biochemical monitoring and reassessment following initiation of hormone replacement therapy.

## Case presentation

A 68-year-old female patient with lung adenocarcinoma was receiving combination therapy with pemetrexed, carboplatin, and pembrolizumab. She had no previous history of pituitary disease, endocrinopathy, head trauma, or cranial irradiation. After completing five cycles of immunotherapy, she presented with progressive lethargy, nausea, reduced appetite, and generalized weakness.

Initial laboratory investigations revealed significant hyponatremia (serum sodium 125 mmol/L) with low serum osmolality (265 mOsm/kg), as shown in Table 1. Endocrine testing demonstrated low morning serum cortisol levels (<27.6 nmol/L) and suppressed thyroid-stimulating hormone with normal free thyroid hormone concentrations, raising suspicion for hypophysitis.

**Table 1:** Serial serum sodium and osmolality measurements

Time Point	Serum Sodium (mmol/L)	Serum Osmolality (mOsm/kg)	Urine Osmolality (mOsm/kg)
Admission	125 ↓	265 ↓	114
Day 3	164 ↑	337 ↑	170
Day 5	159 ↑	331 ↑	305
Pre-desmopressin	149 ↑	299	475
Post-desmopressin	147 ↑	305	569 (28% relative increase after desmopressin)
Day 7	139	298	568
Reference range	135–145	275–295	50–1200

**Figure 1:**
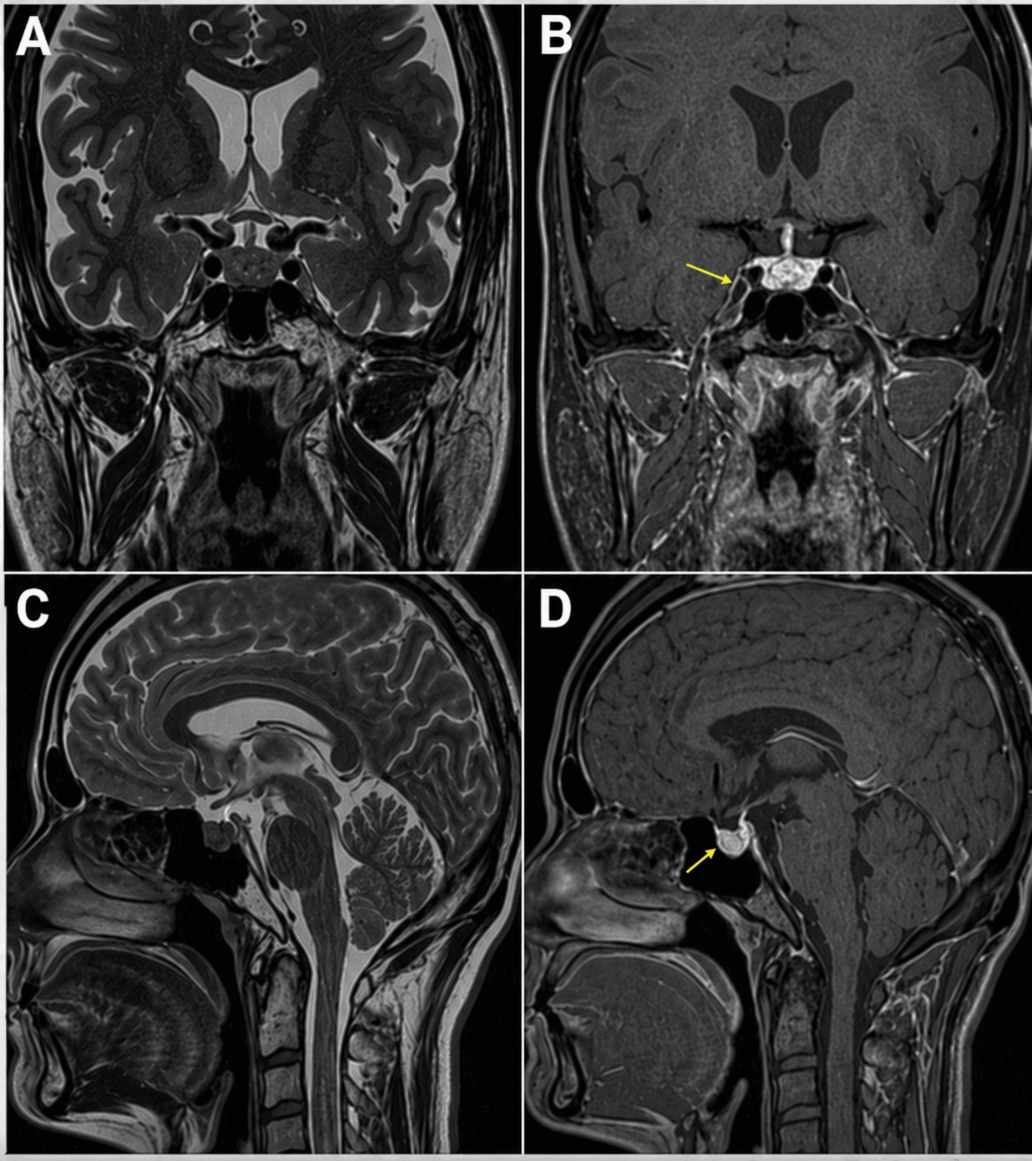
MRI of the brain with pituitary protocol demonstrating immune checkpoint inhibitor–related hypophysitis A. Coronal T2-weighted MRI demonstrating normal appearance of the pituitary gland and stalk. B. Contrast-enhanced coronal T1-weighted MRI demonstrating homogeneous enhancement and expansion of the pituitary gland and stalk (yellow arrow), consistent with hypophysitis. C. Sagittal T2-weighted MRI demonstrating normal size and morphology of the pituitary gland and stalk. D. Contrast-enhanced sagittal T1-weighted MRI showing diffuse enlargement of the pituitary gland with thickening and increased enhancement of the pituitary stalk (yellow arrow), measuring approximately 5 mm, consistent with hypophysitis. MRI, magnetic resonance imaging

Magnetic resonance imaging of the brain with pituitary protocol demonstrated diffuse enlargement of the pituitary gland with thickening of the pituitary stalk, consistent with hypophysitis (Figure 1). Intravenous hydrocortisone and isotonic saline were initiated, resulting in symptomatic improvement.

On the third day of admission, the patient developed abrupt polyuria (as indicated by a marked increase in urine output from a baseline of 1.5-2.0 L/day during admission to 3.7 L/day) accompanied by worsening hypernatremia, with serum sodium peaking at 164 mmol/L. Paired serum and urine osmolality testing revealed inappropriately dilute urine (osmolality of 170 mOsm/kg; Table 1) in the setting of hyperosmolar plasma. A desmopressin challenge confirmed CDI. Serial biochemical trends are summarized in Table 1.

## Discussion

This case illustrates a rare but clinically important coexistence of secondary adrenal insufficiency and CDI in the setting of ICI-induced hypophysitis. While anterior pituitary dysfunction, particularly secondary adrenal insufficiency, is a well-recognized consequence of ICI therapy, posterior pituitary involvement resulting in CDI remains distinctly uncommon [1-3].

Published literature indicates that the majority of ICI-related hypophysitis cases predominantly affect the anterior pituitary, with only a limited number of reported cases demonstrating CDI due to posterior pituitary or pituitary stalk involvement [3-5]. This rarity may contribute to delayed recognition, particularly when initial biochemical abnormalities are dominated by hyponatremia associated with cortisol deficiency [1,2].

The phenomenon of steroid-unmasked CDI has been well described in other clinical contexts, including pituitary apoplexy and structural pituitary disorders, where untreated adrenal insufficiency initially masks renal free-water loss [6,7]. Following glucocorticoid replacement, restoration of renal free-water clearance reveals underlying vasopressin deficiency, leading to abrupt and sometimes severe hypernatremia. Similar mechanisms have been reported in patients with ICI-associated hypophysitis, although such presentations remain rare [4,5,8].

Compared with previously reported cases, the present report is notable for the rapid onset and severity of hypernatremia shortly after steroid initiation. This highlights the dynamic and evolving nature of immune-related endocrine adverse events and underscores the importance of repeated biochemical assessment rather than reliance on initial diagnostic findings alone [1,2].

Recognition of this interaction is critical, as delayed diagnosis of evolving CDI may result in severe electrolyte disturbances and neurological complications. This case adds to the growing body of literature emphasizing the need for heightened vigilance and systematic reassessment of pituitary function in patients receiving ICIs [1,2,8].

## Conclusions

This case highlights the complexity of immune-related endocrine adverse events in patients receiving ICIs. The coexistence of adrenal insufficiency and CDI may lead to diagnostic masking and abrupt electrolyte disturbances following glucocorticoid replacement. Clinicians should maintain vigilance for evolving sodium abnormalities and reassess pituitary function dynamically during treatment. Early recognition and timely initiation of desmopressin are essential to prevent morbidity and ensure safe management of affected patients.
